# The Close Relationship Existing between the Veterinary Profession of To-day and Organized Societies for the Prevention of Cruelty to Animals1Presented to the Convention of Humane Societies at Nashville, October, 1897.

**Published:** 1897-12

**Authors:** W. H. Dalrymple

**Affiliations:** Baton Rouge, La.


### THE CLOSE RELATIONSHIP EXISTING BETWEEN THE VETERINARY PROFESSION OF TO-DAY AND ORGANIZED SOCIETIES FOR THE PREVENTION OF CRUELTY TO ANIMALS.[1]

By W. H. Dalrymple, M.R.C.V.S.,
BATON ROUGE, LA.

I can assure you I feel highly honored by the invitation extended to me, through your local committee, to address this convention of workers in such a noble cause as that of endeavoring to prevent and alleviate the sufferings of nature's dumb nobility.

Statistics with regard to the beneficent results, the world over, of the great and good work of this and kindred associations must be familiar to most of you; consequently, this impressive side of the work I do not propose touching upon, but will confine my remarks chiefly to that important branch of the subject suggested by the caption of my paper.

On account of my devotion to that noble branch of medical science of which I have the honor of being a humble representative, I trust I may be pardoned should my effort, on this occasion, appear to savor somewhat strongly of veterinary professionalism. It is a fact, however, that the modern veterinary profession is as yet but slightly known in many parts of this country, and is in consequence indifferently appreciated. We are constrained to the belief, however, that when the hand of Time has fallen upon its endeavors to right the wrongs of past ages, the dark doings of empiricism, superstition, and cruelty will yield to the photophobic influence of the light of education, reason, and intelligence.

I have been impressed with the idea that the beautiful lines of the poet Burns, parodied to read: "Man's inhumanity to his dumb servitors makes countless thousands mourn," would make a fitting motto for a humane society, and one which should be impressed firmly upon the mind of everyone, but more especially the young and rising generation, because the earlier the impressions made the more lasting are they; not, perhaps, as the outcome of reasoning, but as the result of implicit belief and faith in the maxims taught in early youth.

There can be no doubt that one of, if not the greatest, clogs to the wheels of the progress of the work of our humane societies is ignorance on the part of our people, and indifference, which is born

[1] Presented to the Convention of Humane Societies at Nashville, October, 1897.

of the former; and in order that the acme of our desires may be reached in seeing the day when the physical infirmities of those dumb but noble creatures which God has given us to lighten our daily routine of work, or contribute to our pleasures, have been respected and considered, we must look to education for the accomplishment of this great end.

Where, it might be asked, should this education commence? It is my conscientious conviction that the nursery is the first school in which the seed should be sown, and that the cultivation and fertilization of that seed should be undertaken by every school, whether public or private, in the land. There should be a grounding in physiology, for the purpose of impressing upon the student the normal functions of the various important organs of the animal economy. Attention should also be given to pathology, elementary, if you will, to illustrate the results produced by a deviation from the normal standard of health, and the simpler causes by which these conditions are brought about; a somewhat simple but impressive course of instruction in the physiology and pathology of that system of nerves known as the cerebro-spinal, for the purpose of illustrating the effect of nervous irritability, both direct and reflex; in other words, elucidating the phenomena of pain.

The education of the youth along these lines would, it seems to me, tend to aid very materially the noble work for which our humane societies were organized, and ultimately to decrease the necessity for their existence.

There is another school in which is gained a more intimate knowledge of comparative anatomy, physiology, and pathology that might help along very considerably the humanitarian work. I refer to our medical colleges. It is unfortunate, and I speak it with all due respect, that no little ignorance prevails, at the present day, among members of the medical profession regarding the closeness of the analogy that exists between the two branches of medical science. The result is that but limited assistance to the cause can be expected from that source, not, of course, from lack of desire, but from want of confidence, due to insufficient knowledge of such analogy. If our medical institutions would enlarge their field of teaching in the broad subject of medicine, so as to include comparative studies, of even an elementary character, our practising physicians, armed with a more or less intimate acquaintance with the subject, would then be able to exert a wholesome influence on the public in this regard and give more emphasis and weight to the value of the humanitarian work of our associations.

With regard to the influence the veterinary profession has, or ought to have, in this direction, it might be stated that all graduates of the modern schools are, in virtue of the profession they represent, honorary members of humane societies, if not actually enrolled as such, and are tacitly understood to exert their influence in the cause of humanity in its application to the lower animals.

I claim, then, that the profession of modern veterinary medicine and surgery has the power to be, and in fact is, one of the most potent factors in the education of the people along the lines we have just been considering; and it may be of interest for us to consider, for a few moments, the ground upon which such a claim is based.

Before proceeding with this part of my subject, however, I would like to emphasize the fact that there are many forms of cruelty perpetrated on the lower animals, inadvertently, it may be, and without any cruel intent, but through gross ignorance of the conditions involved. As an illustration of this, I might quote the saying "killed through kindness," and it is true that a great many animals are often subjected to excruciating pain from what might be called "mistaken kindness." For instance, it is a common experience for the veterinarian to be called to relieve the sufferings of some poor animal the victim of over-feeding and its sequelæ: indigestion, irritation, and inflammation of a portion of the alimentary canal. These pain-producing conditions are frequently brought about through mistaken kindness, the result of a want of knowledge regarding the laws of nutrition. It must be borne in mind that there is a limit to the digestive and assimilative powers; and if we, through ignorance or indifference, indulge to excess with food, animals, which have not the intelligence nor the power to say, "Hold, enough," to the point of over-taxing the organs of digestion and producing the agonizing paroxysms we are often compelled to witness, we are, I think, to some extent at least, guilty of cruelty, because such a condition of affairs could be prevented.

Another form of cruelty seen daily on our city streets and thoroughfares, and which we cannot but believe is largely the result of ignorance, is the using of lame horses. Lameness may be defined to be the expression of pain in the act of progression; and as we cannot possibly impeach that noble animal with being guilty of deception, especially to the point of exhibiting signs of pain when not actually in existence, whenever any irregularity of gait is shown it indicates suffering—of course, I allude here to animals that are free from structural deformities—yet we find many people in the enjoyment, seemingly, of unbounded happiness, pleasure-riding behind a poor animal suffering, it may be, very acutely.

Cruelty in another form may be witnessed in many rural sections of the country during the winter season of the year, but which, I opine, escapes the observation, in some measure at least, of the ordinary lay individual. I have been in the position to see and read reports from correspondents of State Departments of Agriculture as to the health and condition of live-stock, and have been overawed at the enormous mortality resulting from actual starvation during the months of winter severity. On almost every report could be seen: "Stock dying from starvation." Now, I do not presume to say that this deplorable state of affairs is occasioned by any positive intention of cruelty, yet I can find no other fitting appellation for it. I claim that anyone possessing an animal, or a number of animals, who does not provide the necessary comfort, in the form of food and shelter, during the extreme seasons of the year, is guilty of an overt act of cruelty; and this also applies where a greater number of animals are kept than can be properly provided with the common comforts of animal life.

There are many other species of cruelty perpetrated on the lower animals, through a lack of education and knowledge, that might be alluded to; but as time will not permit of an exhaustive paper on such an inexhaustible topic, I will be compelled to forego discussion of them on this occasion. The references made may, however, I trust, be the means of awakening special interest among owners themselves with regard to those conditions which inflict forms of cruelty through neglect and indifference, but which may as yet be without the pale of the influence of our humane societies.

(To be continued.)

